# Use of physical restraint in Dutch ICUs: prevalence and motives

**DOI:** 10.1186/cc12331

**Published:** 2013-03-19

**Authors:** RJ Raijmakers, RL Vroegop, H Tekatli, M Van den Boogaard, AW Van der Kooi, AJ Slooter

**Affiliations:** 1 University Medical Center Utrecht, the Netherlands; 2 Radboud University Nijmegen Medical Centre, Nijmegen, the Netherlands

## Introduction

Physical restraints are used to facilitate essential care and prevent secondary injuries. However, physical restraint may be regarded as humiliating. It may lead to local injury and increase the risk of delirium and post-traumatic stress syndrome. Research on physical restraint is scarce. The aim of this study is to investigate the scope of physical restraint use.

## Methods

Twenty-one ICUs ranging from local hospitals to academic centres were each visited twice and 327 patients were included. We recorded characteristics of restrained patients, motives and awareness of nurses and physicians.

## Results

Physical restraint was applied in 74 (23%) patients, ranging from 0 to 54% in different hospitals. Frequent motives for restraint use were 'possible threat to airway' (36%) and 'pulling lines/probes' (31%). Restrained subjects more often had a positive CAM-ICU (34% vs. 16%, *P <*0.001), could less frequently verbally communicate (14% vs. 49%, *P <*0.001), and received more often antipsychotics (49% vs. 28%, *P <*0.001), or benzodiazepines (55% vs. 36%, *P *= 0.003). The use of physical restraint was registered in the patient's files in 48% of cases. Of the 310 interviewed nurses, 258 (83%) were familiar with a physical restraint protocol and 89 (29%) used it in any situation. Thirty percent of the 60 interviewed physicians were aware of the physical restraint status of their patients.

## Conclusion

Physical restraint is frequently used in Dutch ICUs, but the frequency differs strongly between different ICUs. Attending physicians are often not aware of physical restraint use.

